# Minimal climate change impacts on the geographic distribution of *Nepeta glomerulosa*, medicinal species endemic to southwestern and central Asia

**DOI:** 10.1038/s41598-022-24524-8

**Published:** 2022-11-18

**Authors:** Sahar Karami, Hamid Ejtehadi, Hamid Moazzeni, Jamil Vaezi, Maryam Behroozian

**Affiliations:** 1 Quantitative Plant Ecology and Biodiversity Research Lab, Department of Biology, Faculty of Science, Ferdowsi University of Mashhad, Mashhad, Iran; 2 Herbarium FUMH, Department of Botany, Research Center for Plant Sciences, Ferdowsi University of Mashhad, Mashhad, Iran

**Keywords:** Ecology, Plant sciences

## Abstract

Medicinal plants are valuable species, but their geographic distributions may be limited or exposed to extinction by climate change. Therefore, research on medicinal plants in the face of climate change is fundamental for developing conservation strategies. Distributional patterns for a semi-endemic medicinal plant species, *Nepeta glomerulosa,* distributed in southwestern and central Asia was determined based on a maximum–entropy algorithm. We evaluated potential geographic shifts in suitability patterns for this species under two Shared Socioeconomic Pathways scenarios (SSP2-4.5 and SSP5-8.5) of climate change for 2060. Our models based on climatic features indicate that the species occupies montane areas under current conditions; transfer of the model to future climate scenarios indicated that suitable areas for the species will increase in general, and the species will likely track its favored set of climate conditions. But the types and degrees of these changes differ among areas. Our findings can be used to inform conservation management programs for medicinal, endemic, and endangered species that probably respond similarly to climate change in southwestern and central Asia.

## Introduction

Climate change is considered as one of the most important issues globally, with shifting conditions affecting geographic distributions of plant species^1–7^. Studies of various plant species indicate that climate change may reduce the climatically suitable areas for species or shift their geographic distributions^2,6,8,9^. Investigating climate change impacts and the response of wild plant species to these changes is important for effective species conservation and sustainable ecological development^2,9^. An important and long-standing challenge in ecology is understanding the factors limiting geographic distributions, which is especially important in predicting consequences of environmental and climate change for plant species^10–12^.

Numerous modeling approaches have been developed to explore and anticipate future species distributions under changing climates. Ecological niche models (ENMs) are a suite of techniques based on occurrence data and environmental variables that allow researchers to estimate relative suitability of habitats of the species. These approaches can elucidate the relative suitability of sites in areas not occupied by the species, allowing estimates of likely changes in ranges of species over time^13,14^.

**Figure 1:**
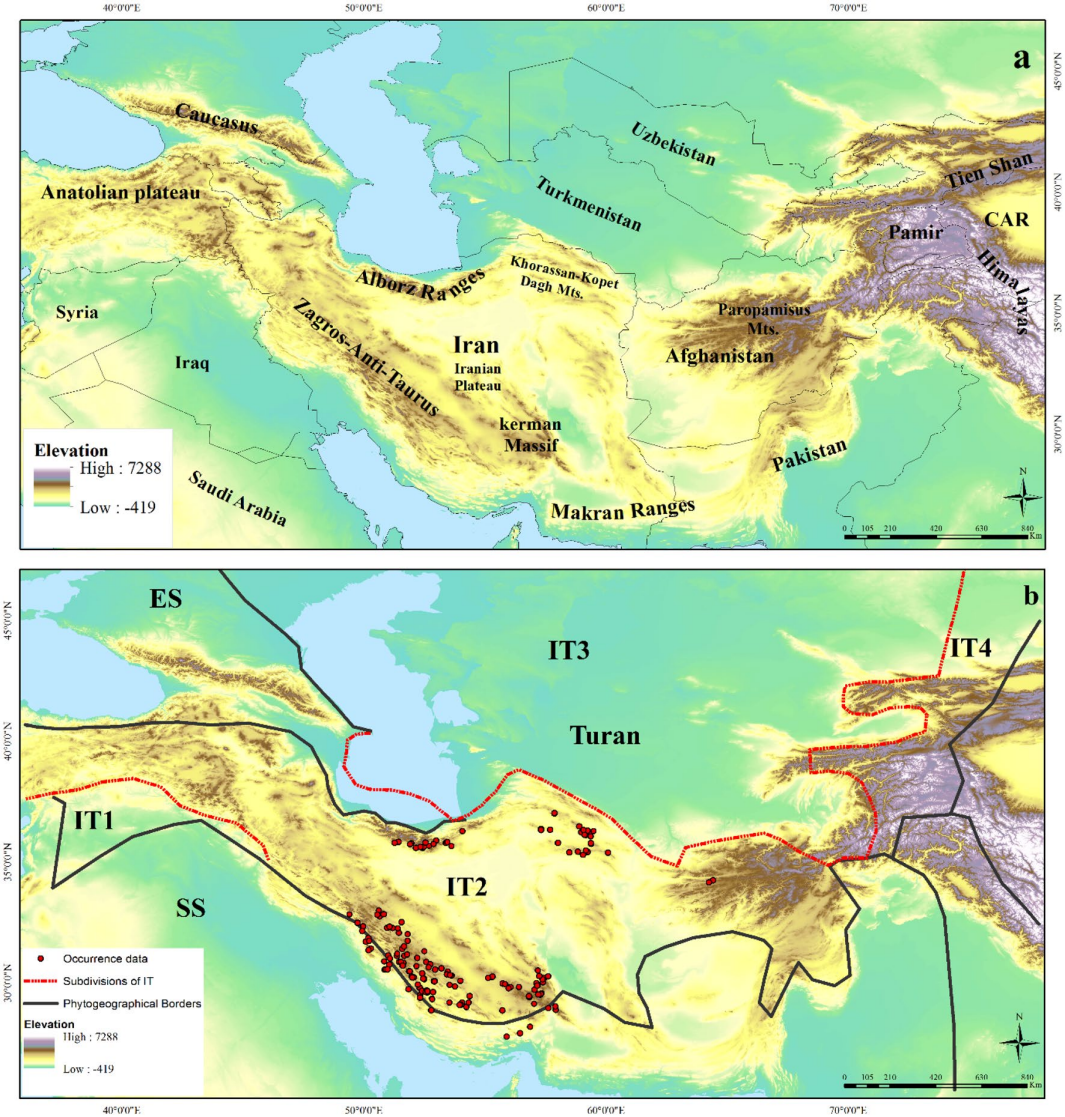
The study area and distribution of *Nepeta glomerulosa* in southwestern and central Asia: (**a**) Topographic map of mountain areas across southwestern and central Asia, (**b**) Phytogeographical borders: IT; Irano–Turanian region, ES; Euro-Siberian region, and SS; Saharo-Sindian region (Phytogeographical borders is taken from Behroozian et al.^2^) (ArcGIS version 10.3.1; http://www.esri.com).

A semi-endemic medicinal plant species of the genus *Nepeta* L. (*Nepeta glomerulosa* Boiss.) was selected for investigating effects of climate change in southwestern and central Asia based on ecological niche models (Fig. 1). *Nepeta* is a large genera, with about 300 species, belonging to Lamiaceae family^15^. It is found across Eurasia, with southwestern Asia and the western Himalayas serving as diversification hotspots^16,17^. *Nepeta* species are used for their antispasmodic, expectorant, diuretic, and antiseptic properties; therefore, they are widely used by pharmaceutics^15–18^.

**Figure 2:**
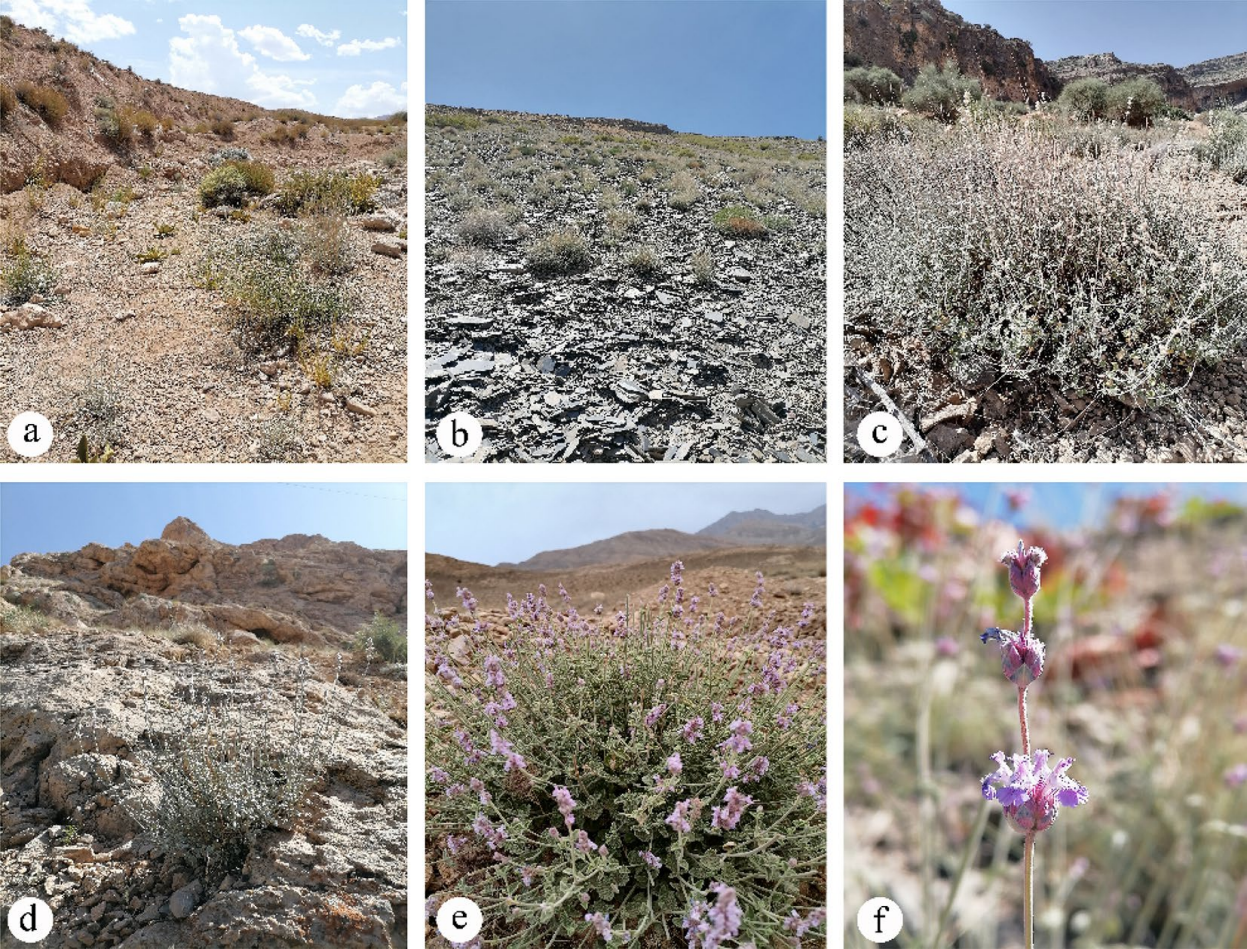
*Nepeta glomerulosa*: (**a**–**d**) Habitats. (**e**–**f**) life form and morphology of flower specifically, photos show (**a**) dry river bed, (**b**) gravelly slopes, (**c**) *Amygdalus* community, (**d**) dry rocky slopes, (**e**) life form, and (**f**) morphology of flower.

*Nepeta glomerulosa* is a semi-endemic species traditionally used to treat pneumonia, itching, and various skin and gastrointestinal disorders^19,20^. Based on taxonomic studies, the species has the problems at the subspecies level and the geographical distributions which makes it important among taxonomists and ecologists^18^. *N. glomerulosa* occurs at elevations of 1500–4000 m in Iran’s Zagros, Alborz, and Khorassan–Kopet Dagh mountains, as well as mountains in Afghanistan^20^ (Fig. 1). The species grows on gravelly and rocky slopes, along with communities of *Artemisia*, *Astragalus*, *Pistacia*, and *Amygdalus*^21^ and also in dry springs and rivers in the Irano–Turanian region (Fig. 2). Previous studies of this species have focused on analysis of their essential oils^22,23^ and hypnotic effects^24^; however, no information has been assembled about the species' geographic distribution or environmental dimensions that shape these distributions. Since *N. glomerulosa* is a semi-endemic medicinal species that is widely used^20,25,26^ underlying likely the effects of climate change on its populations could be useful for conservation planning^2^.

In this study, we studied likely climate change impacts on the distribution of *N. glomerulosa* in southwestern and central Asia under current and modeled future climatic conditions. The aim of this study was to determine key environmental factors limiting the distribution of the species. We used this information to estimate the likely change in the distribution of *N. glomerulosa* coming decades, and assessed how conservation efforts can shift in response.

## Materials and methods

### Study area

The Irano-Turanian (IT) region is one of the most important phytogeographic zones, covering a large swath of southwest and central Asia. IT is divided into four sub-regions (IT1, IT2, IT3, and IT4) (Fig. 1), with IT2 serving as the main center of speciation and endemism, with low annual precipitation (low in winter and high in summer, in some areas), low winter temperatures, and a high continentality index^27,28^. *Nepeta glomerulosa*, as a species of the IT region, is distributed in the Zagros and Alborz Mountain ranges, Khorassan-Kopet Dagh Floristic Province in Iran and the Paropamisus Mountains in Afghanistan (IT2). This species has also been seen as transitional between two regions the IT and the Sahara–Sindian in the southern and southwestern parts of the country^18^ (Fig. 1).

### Occurrence data

We obtained occurrence data from the following herbaria: FUMH, HUI, SFAHAN, HSHU, MIR, Natural Resources of Khuzestan, Animal & Natural Resources Research Center of Hormozgan, and Natural Resources of Kohgiluyeh and Boyer–Ahmad (Table S1). We also used Global Biodiversity Information Facility (http://www.gbif.org; GBIF.org (20 February 2021) GBIF Occurrence Download https://doi.org/10.15468/dl.2pkbuk).

Occurrence data were reviewed and filtered in two steps. First, we checked carefully occurrence data for localities falling outside the species’ known range by Google Earth, and deleted them in consultation with the herbarium expert. Second, one pair of records less than ~ 1 km apart was removed to match the resolution of the climatic variables using the spThin package^29^ in R (version 4.1.1 ).

### Climate data

For current and potential future situations, a set of bioclimatic variables was established. The 19 bioclimatic variables for current conditions were acquired from WorldClim version 1.4 (http://www.worldclim.org), at a spatial resolution of 30" (~ 1 km). Four of the layers (bio 8, bio 9, bio 18, and bio 19) were removed because they include known spatial artifacts^30–32^. To avoid highly correlated variables, we used Pearson correlation coefficients^33^ in R. Then, we removed one of each pair of variables with a correlation ≥ 0.8 based on our knowledge of variable importance in the ecological conditions and habitat of the species. The remaining seven variables included bio 2, bio 3, bio 7, bio 10, bio 12, bio 15, and bio 17.

Bioclimatic variables under future climate scenarios were downloaded from WorldClim website (https://www.worldclim.org/data/cmip6/cmip6_clim30s.html) at a spatial resolution of 30". Nine general circulation models (GCMs) were selected under two SSP2-4.5 and SSP5-8.5 scenarios, including (1) ACCESS-CM2, (2) BCC-CSM2-MR, (3) CMCC-ESM2, (4) GISS-E2-1-G, (5) HadGEM3-GC31-LL, (6) IPSL-CM6A-LR, (7) MIROC6, (8) MPI-ESM1-2-HR, and (9) MRI-ESM2-0. This variety of GCMs was used to illuminate the uncertainty in predictions of the potential distribution of the species in the future^33^.

### Ecological niche modeling

Models were performed using Maxent version 3.3.1^34^. Maxent is one of the valuable methods that estimates the functions to represent the environmental variables and habitat suitability to approximate geographical distribution of species^14^. We applied “Model selection” approach using the ENMeval package^35,36^ in R. A set of 22 regularization parameter values (0.1, 0.2, 0.3, 0.4, 0.5, 0.6, 0.7, 0.8, 0.9, 1; 1.25, 1.5, 1.75, 2; 3, 4, 5, 6, 7, 8, 9, and 10) and 31 combinations of model response types (linear, quadratic, product, threshold, and hinge responses) were explored. Regularization parameter and the combinations of response types are features needed for Maxent modeling^3,14^. Out of the 682 candidate models, the best model was chosen based on the lowest value of the Akaike information criterion (AICc) to identify the model of suitable area and conditions consistent with the data^36^.

The first model was created using maximum entropy algorithm (Maxent), with 10,000 pseudo-absences and 10 cross-validation replicates among occurrence data based on seven variables (bio 2, bio 3, bio 7, bio 10, bio 12, bio 15, and bio 17) (Tables S2 and S3); it was performed across southwestern and central Asia under the current conditions. Since the contribution percentage of bio7 and bio15 = 0 (Table S2), we removed them from the analysis and the final model was run again based on five variables (bio 2, bio 3, bio 10, bio 12, and bio 17) (Table S4). We also transferred the final model to future conditions allowing extrapolation and clamping. When models projected on one landscape are fitted on another, the new landscape may include environmental conditions beyond the range of conditions observed in the appropriate landscape. In this case, the response curve may be closed beyond the range of the fitted data by setting it to a constant value equal to the predicted value at the edge of the range^32^. Median values across the replicate versions of the final model were used to estimate suitability across the region for current conditions. The median of all of the medians for the 9 GCMs was calculated to interpret the future potential geographic distribution of *N. glomerulosa* under different climate change scenarios (i.e., the two SSPs). For assessing the model’s performance, records split ten times (cross-validation) for calibrating the model and estimating the precision of model prediction^37^. Based on the range of values across the 10 cross-validation replicates, we estimated uncertainty for current and future conditions. Suitability scores were converted to binary using maxSSS (maximizing the sum of sensitivity and specificity), one of the best threshold selection methods for presence and absence data and also large samples^38^. The potential distribution of *N. glomerulosa* from current conditions to SSP2-4.5 and SSP5-8.5 conditions estimated based on the binary map and threshold value in ArcGIS by special analysis tools and reclassifying raster layers in ArcGIS version 10.3.1 (http://www.esri.com).

## Results

We obtained a total of 384 occurrence records for *N*. *glomerulosa*. Of these records 202 were eliminated as they were repetitive or lacked adequate precision. To reduce autocorrelation among records, 11 additional records were also removed under the 1 km distance criterion. In the end, the model was calibrated and evaluated based on 171 records, representing the whole set of data available on the species' occurrence.

Using correlations among environmental variables, the variables bio 1, bio 4, bio 5, bio 6, bio 10, bio 11, bio 13, bio 14, and bio 16 were omitted (Table S2). The final model was performed with five variables: mean diurnal range (bio 2), isothermality (bio 3), mean temperature of warmest quarter (bio 10), annual precipitation (bio 12), and precipitation of driest quarter (bio 17) (Table S4). Two variables (bio 2 and bio 12) explain > 93% of overall contribution of variables, and bio 12 was the most important variable in our study (Table S4, Figs. S1, and S2).

In total, 682 models were evaluated in the process of model selection. The best model for five variables, included only linear, quadratic, and product features and a quite low regularization parameter of 0.1. The model had a high AUC value (0.94) (Fig. S3) that discriminates properly between presences and absences, and it has good power to estimate potential distributions of *N. glomerulosa* under present-day condition and different future scenarios.

**Figure 3:**
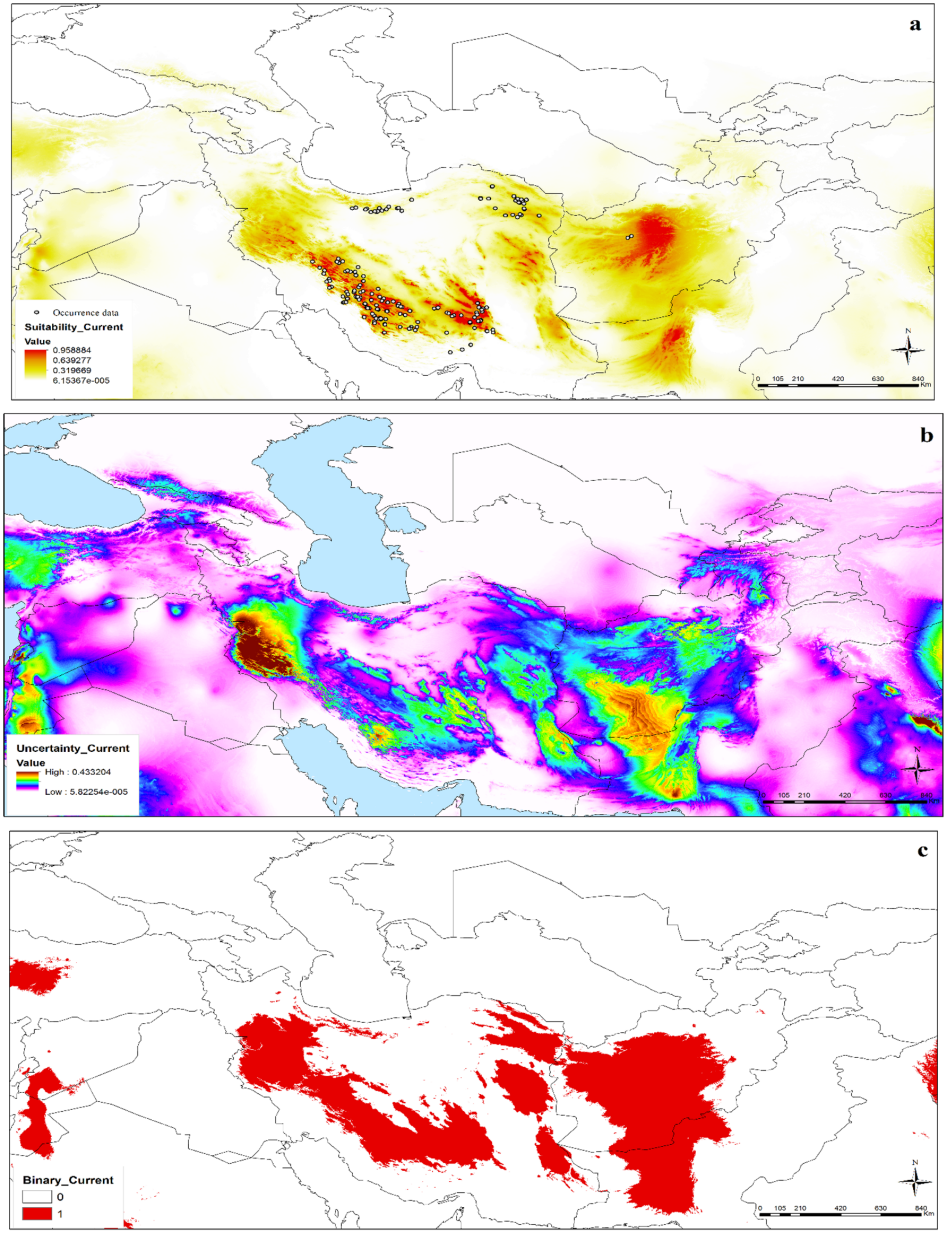
Predicted currently suitable regions for *Nepeta glomerulosa* across the southwestern and central Asia: (**a**) median prediction (based on median values in the replicates of the final model for current conditions), (**b**) uncertainty map (range through 10 cross–validation replicates), and (**c**) binary map (based on suitability scores and maxSSS threshold) (ArcGIS version 10.3.1; http://www.esri.com).

Models indicated suitable conditions with high confidence for present-day conditions for *N. glomerulosa* throughout the montane regions of Iran, Afghanistan, and Pakistan. Highest suitability in Iran was in the Zagros Mountains, which extend from west to south, as well as in the Paropamisus Mountains in Afghanistan, and a small part of southern Pakistan (Fig. 3). The same areas were predicted based on the binary map and thresholding approach (Fig. 3c).

**Figure 4:**
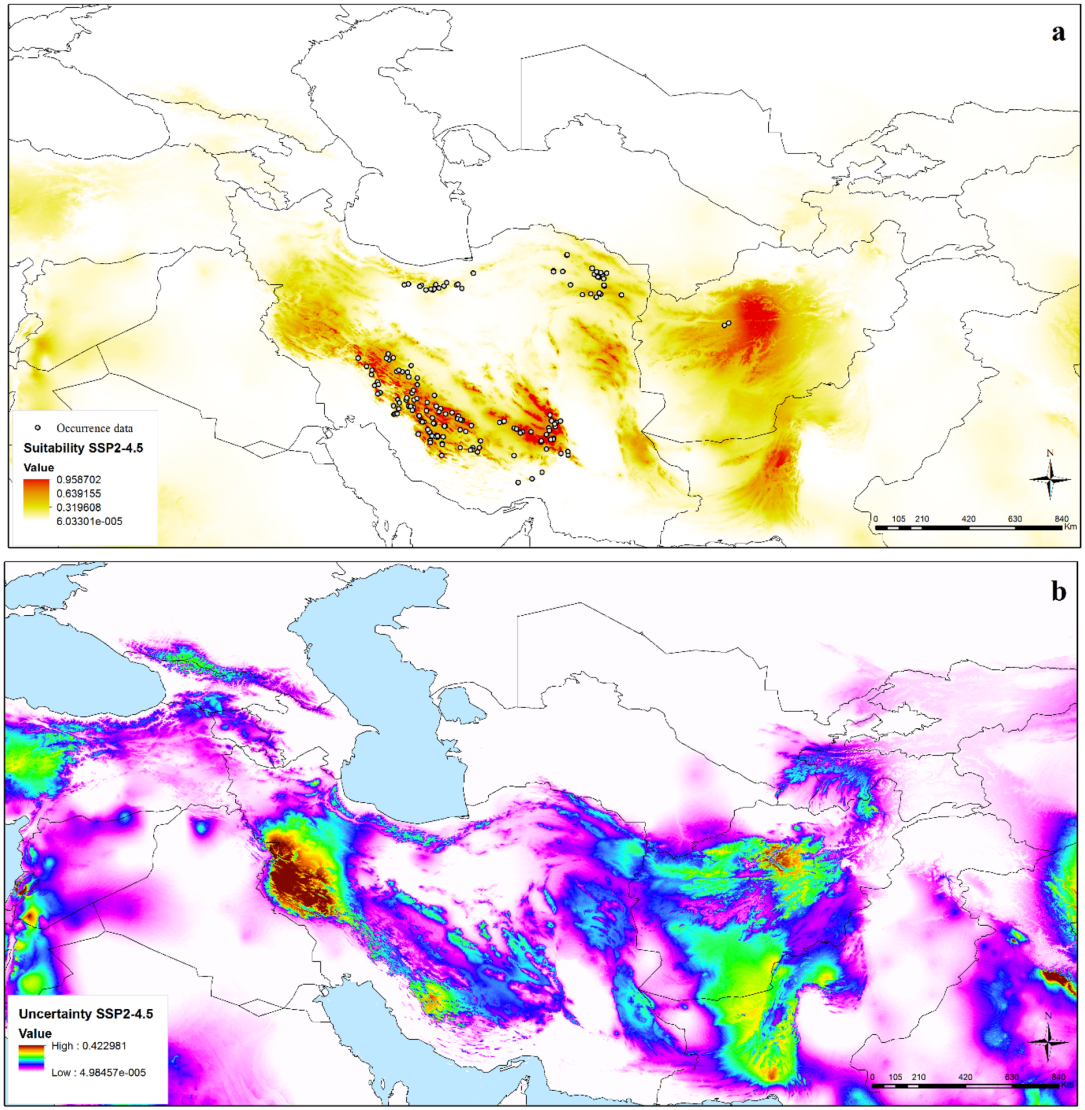
Potential future distribution of *Nepeta glomerulosa* under Shared Socioeconomic Pathways (SSP2-4.5) across the southwestern and central Asia: (**a**) median prediction, (**b**) uncertainty map (ArcGIS version 10.3.1; http://www.esri.com).

**Figure 5:**
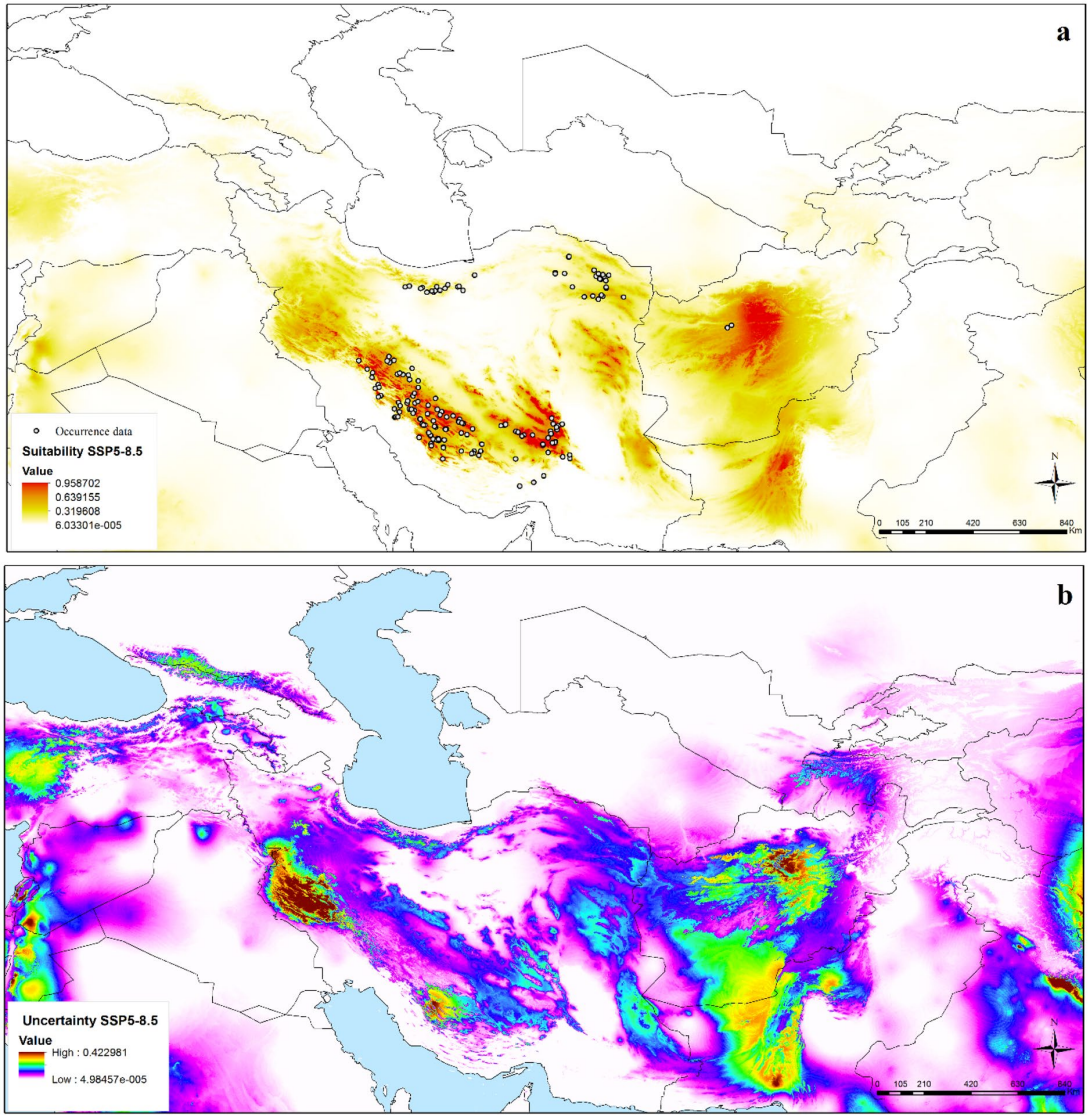
Potential future distribution of *Nepeta glomerulosa* under Shared Socioeconomic Pathways (SSP5-8.5) across the southwestern and central Asia: (**a**) median prediction, (**b**) uncertainty map (ArcGIS version 10.3.1; http://www.esri.com).

With the transfer of the model to future climate scenarios, an overall distributional pattern similar to current distributions was identified. The future potential distribution under SSP2-4.5 was concentrated in the Zagros Mountains, Kerman Massif, montane parts of eastern Iran (southern Khorassan and Sistan); the Paropamisus Mountains in Afghanistan, and the Bruhui Range in Pakistan (Figs. 4, S4a). Model predictions under SSP5-8.5 also focused in the Zagros Mountains, Paropamisus Mountains in Afghanistan, and southern Pakistan as highly suitable areas (Figs. 5, S4b). High uncertainty was observed in western Iran and small parts of southern Afghanistan, Pakistan and India under present-day conditions (Fig. 3). The western part of Iran and small parts of central Afghanistan, southern Pakistan and Syria and west of India showed high uncertainty under SSP2-4.5 (Fig. 4). High uncertainty areas under SSP5-8.5 matched those under SSP2-4.5 (Fig. 5). Uncertainty under SSP2-4.5 and SSP5-8.5 is increased compared current condition. Based on the binary map and threshold value by special analysis tools and reclassifying raster layers in ArcGIS, from current conditions to SSP2-4.5 and SSP5-8.5 conditions, the potential distribution of *N. glomerulosa* increased by 2.5 and 1.7% respectively.

## Discussion

Ecological niche models are frequently used to describe potential distributions of endangered, endemic, and medicinal species^2,39–41^. Here the potential distribution of *N. glomerulosa,* a semi-endemic medicinal plant species, was estimated across southwestern and central Asia under current and future climate conditions. Annual precipitation (bio12) and Mean diurnal range (bio 2) were the most important explanatory climatic variables and account for 64.9 and 29%; respectively, based on five variables (Table S4). When bio12 is < 130 mm, the existence probability of *N. glomerulosa* is 0; and then it increases with the continuous increase of bio12. Finally, the existence probability can reach more than 0.9 with bio12 = 180 mm. Bio2 is considered as a temperature-related climatic variables. Based on our results, the existence probability of *N. glomerulosa* decreases with bio2 more than 50 °C and it is close to 0 with the continuous increase of bio 2. The stable temperature is 25 °C (Fig. S1). The importance of Annual precipitation and Mean diurnal range have been confirmed in previous study at montane areas in Irano-turanian region^2,7^. Annual precipitation also was the most important bioclimatic drivers at a regional scale in central Iran and as the second influential climatic factor in west Iran^1,5^.

A deep link exists between plant distributions and climatic factors. The Mediterranean macrobioclimate is dominant in the IT biogeographic region^27,42^. Three Mediterranean bioclimatic classifications occur in areas where *N. glomerulosa* occurs: (1) Mediterranean pluviseasonal-continental (Mpc) with much annual precipitation during winter months in the Zagros mountains and parts of the Alborz, Kopet-Dagh and Allah Dagh-Binalud mountains^27,43^; (2) Mediterranean xeric-continental (Mxc) with summer drought and low total annual precipitation in western and southwestern Iran, most of the Kopet-Dagh Mountains, and parts of the Kerman Massif; and (3) Mediterranean xeric-oceanic (Mxo), with relatively long summer droughts, low annual precipitation, but relatively elevated average winter temperature minima on the southern flanks of the Kerman Massif in southeastern Iran^27^ (Fig. 1). According to a recent study, the highlands of Afghanistan have low annual temperature ranges: the maximum average summer temperature does not exceed 15 °C, and average winter minimum temperatures are below zero^44^. Despite annual temperature ranges (bio15) can be an effective factor in Afghanistan, it does not contribute based on our results in the study area. In Afghanistan, rainfall is rare, with maximum amounts falling in the northern highlands in March and April^44^. Given the relative lack of accurate information on climatic conditions Afghanistan, we focus on bioclimatic regions manifested in IT2 in Iran (Fig. 1).

Transferring the model to future climate scenarios, an overall distributional pattern similar to current distributions was identified, such that *Nepeta glomerulosa* species occupies the same suitable habitats under current and future conditions. However, our results show an increase with the potential distribution of species under SSP2-4.5 and SSP5-8.5 scenarios (2.5 and 1.7%). The uncertainty under the future conditions is reduced compared to the current conditions (Figs. 3, 4 and 5). It seems that the *N. glomerulosa* species follows its favorable conditions under the effect of climate change. A similar pattern can be seen in the previous study in the Irano-Turanian region^2^.

Given the distribution and presence of the species in the elevational range of 1500–4000 m, both the effect of precipitation at low elevations and the effect of temperature at high elevations^45–47^ are considered important factors in montane areas. Climate parameters, particularly temperature, were recently identified as crucial in limiting plant dispersal on local-to-regional scales in montane ecosystems^2^. One factor that can have a significant impact on distributions of species is dispersal^48^. Although no information is available on how seeds are dispersed in *N*. *glomerulosa*. However, based on our field observations, *N*. *glomerulosa* has a small population size because individuals are clumped and are well-separated from other populations. Based on previous studies, there are a number of dispersal mechanisms in lamiaceae (*Salvia* L.) such as; dispersal by water (hydrochory), dispersal by wind (anemochory), dispersal by animals (zoochory), dispersal by ants (myrmecochory), dispersal by gravity (barochory)^49^. Anemochory is also particularly common in Labiates in arid regions^50^ and myrmecochory is a well-known mechanism in *Lamium amplexicaule* L. species^51^. Therefore, these mechanisms may affect *N*. *glomerulosa* distribution across landscapes, but information is scanty.

Climate change ranks among the most powerful elements influencing future distributions of suitable areas for species. Human activities also have significant impacts on distributions of plants^52^. Since *N. glomerulosa* considered as a medicinal species and has small population sizes, human activities (e.g., irregular harvesting, heavy grazing, urbanization and suburbanization) can reduce its distribution in the future. These factors, their interactions and other potential influences (e.g., invasive species) may affect the distribution of *N. glomerulosa* into the future.

## Conclusions

We investigated the potential geographic distribution of *N. glomerulosa* under current and future climate conditions using ecological niche modeling methods. Regions with high suitability were concentrated in montane areas, and climate change may increase the species’ potential distributional area on regional scales. Although this species occupies a diversity of elevations, climate change effects will likely allow the species track its favored set of climate conditions. However, factors such as excessive harvesting by humans, development, and overgrazing may reduce the habitable geographic areas of the species because it has small, isolated populations. Future studies should assure detailed sampling of different populations under diverse conditions, assessment of the species, interactions with other plants, study of effects of ecological and edaphic factors on different populations in different areas, assessment of mechanisms of seed dispersal and evaluation of possible barriers that may cause genetic and geographic isolation of populations.

## Supplementary Information

Supplementary Information.

## Data Availability

The datasets used during the current study available from the corresponding author on reasonable request.
