# Supplementary material for: Minimal climate change impacts on the geographic distribution of Nepeta glomerulosa, medicinal species endemic to southwestern and central Asia
Source: Sci Rep. 2022 Nov 18;12:19893. doi: 10.1038/s41598-022-24524-8 (PMC9674666; doi:10.1038/s41598-022-24524-8)
Supplement: Supplementary file 1 — Supplementary Information. [file 41598_2022_24524_MOESM1_ESM.docx]

**Supplementary Information (SI)**

**Minimal climate change impacts on the geographic distribution of *Nepeta glomerulosa*, medicinal species endemic to southwestern and central Asia**

Journal: *Scientific Reports*

**Sahar Karami, Hamid Ejtehadi^*^, Hamid Moazzeni, Jamil Vaezi, Maryam Behroozian**

Corresponding author:

E–mail: hejtehadi@um.ac.ir

Address: Quantitative Plant Ecology and Biodiversity Research Lab, Department of Biology, Faculty of Science, Ferdowsi University of Mashhad, Mashhad, Iran.

**Supplementary Table S1.** Occurrence data used in analyses

| **Area** | **Latitude** | **Longitude** | **Elevation** | **Herbarium number** | **Herbarium name** |
| --- | --- | --- | --- | --- | --- |
|  |  |  |  |  |  |
| Khorasan, S Chenaran, Dolat Abad, Baz-e Siah Sang | 36˚ 18' 55.4" | 59˚ 05' 29.1" | 2180 | 44515 | FUMH |
| Khorasan, Chenaran, 1km Darame to Ferizi | 36˚ 29' 37.5" | 58˚ 53' 00.3" | 1964 | 36796 | FUMH |
| Khorasan, NW Torbat-e-Jam, Bardu village, Ors forest | 35˚ 24' 28.8" | 60˚ 04' 26.9" | 1625-1750 | 43434 | FUMH |
| Khorasan, Torbat Heydariyeh, RudMajan village | 35°26'46.16" | 58°50'3.10" | 1750 | 15609 | FUMH |
| Khorasan, west of Torbat Heydariyeh, Sorkh Abad | 35°18'52.42" | 59° 2'41.65" | 1400 | 15319 | FUMH |
| Khorasan, Sabzevar, between Saroogh and Kuh-e Nazargah, | 36˚ 23' 24.6" | 57˚ 18' 45.3" | 2482 | 42721 | FUMH |
| Khorasan, Mashhad, Dehbar, Zou peak | 36°17'9.66" | 59°28'42.45" | 1800 | 10526 | FUMH |
| Khorasan, Mashhad, Dehbar | 36°14'28.93" | 59°16'50.70" | 1800 | 10519 | FUMH |
| Khorasan, E Neyshabur, Garine | 36° 7'44.20" | 59° 9'51.16" | 1900 | 27769 | FUMH |
| Khorasan, W Mashhad, Kang, Binalud mountian | 36°18'45.51" | 59°14'29.44" | 2100 | 27628 | FUMH |
| Khorasan, NW Mashhad, Golmakan, Cheshme Sabz | 36°20'47.93" | 59° 3'47.48" | 1560 | 13261 | FUMH |
| Khorasan, Esfarāyen, Kūh-e Shāh Jahān | 37° 2'51.55" | 57°52'8.57" | 2750 | 24333 | FUMH |
| Khorasan, W Sabzevar, east of Saroogh mountain | 36˚ 20' 31.6" | 57˚ 18' 43.3" | 1590-1650 | 42443 | FUMH |
| Khorasan, Mashhad, Pivehjan, Binalud mountain | 36° 4'10.36" | 59°21'8.43" | 2100 | 22340 | FUMH |
| Khorasan, S Mashhad, Robat Sefid | 35°47'56.28" | 59°21'59.48" | 1700 | 13671 | FUMH |
| Khorasan, Torbat Heydariyeh, Kameh Olia | 35°29'2.04" | 59°10'12.46" | 1700 | 22188 | FUMH |
| Khorasan, easth of Neyshabur | 36˚ 07' 52.9" | 59˚ 06' 37.5" | 1850-1900 | 46385 | FUMH |
| Khorasn, 50 km NE Kashmar, Kuh-e Bezq | 35°24'34.99" | 58°29'6.91" | 1900-2500 | 35783 | FUMH |
| Khorasan, 30 km north of Torbat Heydariyeh | 35°26'14.70" | 59°14'33.72" | 1900 | 21317 | FUMH |
| Khorasan, Neyshabur, Bojan, Mount Binalud | 36°14'56.95" | 58°58'1.46" | 1500-2700 | 48952 | FUMH |
| Khorasan, Esfarāyen, Kūh-e Shāh Jahān | 37° 1'28.17" | 57°51'32.79" | 1400-2500 | 48620-48576 | FUMH |
| Khorasan, 18 km from Sabzevar to Esfarāyen | 36°21'9.34" | 57°41'18.63" | 1600 | 48400 | FUMH |
| Khorasan, 28 km south of Sabzevar | 35°48'54.72" | 58° 0'27.16" | 1500 | 21232 | FUMH |
| Khorasan, south of Robat Sefid | 35°45'47.39" | 59°22'28.18" | 1700 | 80482 | FUMH |
| Esfahan, Semirom, Abmalakh | 31° 8'0.93" | 51°21'16.86" | 2000-2600 | 31388 | HSHU |
| Fars, Shiraz, Bamou park | 29°42'11.56" | 52°36'9.36" | 2700 | 24325 | HSHU |
| Khuzestan, Dehdez, Kuh-e Sefid | 32°10'10.50" | 49°55'38.69" | 2300-2750 | 74521 | HSHU |
| Yazd, Herat, Chenar Naz | 30° 5'35.49" | 53°58'49.10" | 2340 | 1924 | HSHU |
| Fars, Marvdasht, Sivand river | 30° 4'42.15" | 52°54'36.76" | 1580 | 66912 | HSHU |
| Fars, Firooz Abad, Maymand, Kuh-e Sefid dar | 28°52'59.05" | 52°46'47.65" | 1750-2850 | 71420 | HSHU |
| Fars, Fasa, Kherman Kuh | 29°12'8.66" | 53°31'57.16" | 2400-2650 | 46848 | HSHU |
| Fars, 20 km from Estahban to Neyriz, south of Bakhtegan lake | 29°12'6.47" | 54° 2'57.01" | 1750 | 47085 | HSHU |
| Fars, south of Estahban, Kuh-e Bash | 29° 6'47.19" | 54° 0'48.37" | 1700-2200 | 46979 | HSHU |
| Fars, south of Estahban, Kuh-e Toodeh | 29° 6'31.27" | 53°56'51.95" | 1350 | 7378 | HSHU |
| Fars, Shiraz, Darengun, Kuh-e Dali | 29°24'19.71" | 52°19'17.82" | 1820-2200 | 83640 | HSHU |
| Fars, 60 km south of Abadeh, Kuh-e Bel | 30°47'18.29" | 52°45'2.78" | 2750 | 17346 | HSHU |
| 6 km SE Shul | 29°56'29.57" | 52°13'18.41" | 2400 | 6738 | HSHU |
| Fars, Shiraz, Kuh-e Bamou | 29°41'43.82" | 52°35'3.95" | 2200 | 317 | HSHU |
| Fars, Shiraz, Baba Kuhi | 29°38'30.10" | 52°32'59.87" | 1709 | 316 | HSHU |
| Yasuj, Kuh-e Dena, northern slope | 30°50'10.66" | 51°38'18.27" | 2500 | 46051 | HSHU |
| Fars, 40 km in Shiraz to Fasa road, Maharlu lake | 29°20'0.09" | 52°48'58.47" | 1604 |  | HSHU |
| Fars, Sivand, Kuh-e Kale badi | 30° 05' 07.9" | 52° 55' 35.5" | 1830 | 243 | HSHU |
| Kerman, 25 km to north of Shahrbabak, Abdar | 30° 15' 00.63" | 55° 17' 10.75" | 2277 | 390 | HSHU |
| Kerman, 25 km to north of Shahrbabak, Kuh-e Lakona | 30°15'0.72" | 55°17'9.32" | 2420 | 249 | HSHU |
| Kerman, 25 km to north of Shahrbabak, Abdar | 30 °16' 11.60" | 55° 17' 52.10" | 2353 | 355 | HSHU |
| Kerman, 25 km to north of Shahrbabak, Kuh-e Lakona | 30 °16'22.60" | 55° 18' 23.60" | 2415 | 169 | HSHU |
| Fars, 15km SW Eqlid, Tang-e Esar | 30° 53' | 52° 34' | 2570 |  | HSHU |
| Fars, 24 km SE Estahban, Kuh-e Bash | 29°01' | 54° 14' | 1590 |  | HSHU |
| Fars, 8 km NE Abade Tashk , near to Koromine mine | 29°52' 7.92" | 53° 45' 46.8" | 2080 |  | HSHU |
| Fars, Dasht-e Arzhan , Mard abad village, Kuh-e Tashk | 29° 45' | 51° 50' | 2150 |  | HSHU |
| Fars, 75 km SW Eghlid, Sade | 30° 42' | 52° 10' | 2200 |  | HSHU |
| Fars, Sepidan | 30°16' | 51° 59' | 2300 |  | HSHU |
| Fars, Abade, Simakan | 30° 25' | 53° 26' | 2500 |  | HSHU |
| Fars, Dehbid | 30°37'0.46" | 53°12'11.74" | 2300 |  | HSHU |
| Fars, 50 km S Eqlid, Khunisht | 30°39'34.87" | 52°40'41.09" | 2320 |  | HSHU |
| Fars, 22km NW Marvdasht, Kuh-e Ayoob | 30° 2'55.54" | 52°39'7.36" | 1900 |  | HSHU |
| Fars, 9 km SW Jeyan, Dareshkaft | 30° 28' 57.84" | 53° 28' 11.76" | 2700 |  | HSHU |
| Fars, Shiraz, Kuh-e Bamou | 29°.4' | 52°.53' | 1530 | 13 | HSHU |
| Kerman, Sirjan, Kuh-e Panj | 29° 50' 50.08" | 56° 2' 54.64" | 2717 | 20 | HSHU |
| Fars, 5 km SW Sepidan | 30° 16' 57.61" | 51° 57' 27.91" | 2387 |  | HSHU |
| Yasuj, Sisakht | 30° 52' 21.86" | 51° 27' 54.41" | 2431 |  | HSHU |
| Fars, Marvdasht, Sad-e Droud zan | 30°11'43.25" | 52°26'40.71" | 1654 | 231 | HSHU |
| Fars, Bavanat, Koupan | 30° 18' 54.60" | 53° 37' 18.70" | 2654 |  | HSHU |
| Fars, Shiraz, Kuh-e Drak | 29° 40' 22.44" | 52° 25' 52.25" | 2028 |  | HSHU |
| Fras, Sepidan. Kahkaral village | 30° 14' 25.98" | 52° 3' 41.35" | 2425 |  | HSHU |
| Fars, Shiraz to Kharameh, Dasht kuh | 29° 37' 2.54" | 52° 36'48.89" | 1786 | 18 | HSHU |
| Kerman, Sarcheshmeh, Mani | 29° 59'32.31" | 55° 43'22.95" | 2428 |  | HSHU |
| Fars, Shiraz, kuh-e Sabz pooshan | 29° 21'58.86" | 52° 48'13.38" | 1736 |  | HSHU |
| Fars, Nayriz, 18 km W Meshgan | 29° 29'15.60" | 52°18'36.28" | 2314 |  | HSHU |
| Fars, west of Sepidan | 30° 15'9.68" | 51° 59'32.64" | 2235 |  | HSHU |
| Fars, north of Abadeh, Shams abad | 31° 19' 18" | 52° 29'34.33" | 2258 |  | HSHU |
| Fars, Fasa, Emamzade Esmaeil | 29° 82'1.62" | 53° 25'30.87" | 1702 |  | HSHU |
| Fars, Eqlid, Bakan | 30° 24'41.65" | 52° 24'22.25" | 2219 |  | HSHU |
| Fars, Shiraz, Kuh-e Drak | 29° 40'21.17" | 52° 25'52.27" | 2091 |  | HSHU |
| Fars, Shiraz, Kuh-e Drak | 29° 40'10.40" | 52° 25'38.82" | 2261 |  | HSHU |
| Fars, Shiraz, Ghalat | 29° 48'24.03" | 52° 18'43.30" | 2325 |  | HSHU |
| Kerman, Bidkhab | 29° 47'41.69" | 55° 58'36.17" | 2365 |  | HSHU |
| Fars, Sepidan, Komehr | 30° 29'46.75" | 51° 51'41.47" | 2456 |  | HSHU |
| Fars, Marvdasht, Sivand | 30° 34'2.87" | 52° 56'14.26" | 1804 |  | HSHU |
| Fars, Shiraz, Ghalat | 29° 50'21.81" | 52° 16'41.54" | 2334 |  | HSHU |
| Fars, Shiraz, Bamou park | 29° 38'10.94" | 52° 50'49.89" | 1668 |  | HSHU |
| Fars, Marvdasht, Sivand | 30° 05'09.17" | 52° 52'59.49" | 1885 |  | HSHU |
| Fars, Marvdasht, Behesht-e Gomshodeh | 30° 20'11.79" | 52° 93'6.83" | 1877 |  | HSHU |
| Fars, Shiraz, Ghalat | 29° 50'24.23" | 52° 17'22.76" | 2289 |  | HSHU |
| Fars, Shiraz, Bamou park | 29° 37'43.85" | 52° 37'33.64" | 1932 |  | HSHU |
| Fars, Shiraz, Drak | 29° 40'16.21" | 52° 25'44.36" | 2200 |  | HSHU |
| Fars, Marvdasht, Road of kushk to Dorood Zan | 30° 09'47.88" | 52° 29'33.36" | 1647 |  | HSHU |
| Fars, Neyriz, Meshgan, shahrak-e Shahed | 29° 28'16.48" | 54° 21'40.65" |  | 90 | HSHU |
| Fars, Shiraz, Gouyom | 29° 40'32.21" | 52° 23'45.17" | 1847 |  | HSHU |
| Fars, Abadeh, Koular | 31° 02'52.05" | 52° 11'53.15" | 2427 |  | HSHU |
| Fars, Sepidan, Bereshne | 30° 14'42.98" | 51° 59'52.86" | 2205 |  | HSHU |
| Fars, Neyriz, west of Jam asb | 29° 12'04.47" | 54° 19'17.70" | 2254 | 73 | HSHU |
| Kerman, Sirjan, Kuh-e Panj | 29° 50'36.91" | 56° 24'8.59" | 2685 | 10 | HSHU |
| Fars, Arsenjan, tang-e Ashkan, Kuh-e Khom | 29° 55'55.30" | 53° 32'21.20" | 1874 | 254 | HSHU |
| Kerman, Kuh-e Nasr | 30°13'5.49" | 57°26'4.73" | 3100 |  | HSHU |
| Kerman, Kuh-e Lalezar | 29.436667 | 56.625 | 3000 |  | HSHU |
| Esfahan, Fereydun Shahr, Damaneh | 33° 00' 26" | 50° 37' 11" | 2455 | 17084 | HUI |
| Esfahan, Falavarjan, Shah Jahan | 32°32'3.82" | 51°33'2.86" | 1900 | 16732 | HUI |
| Esfahan, Chadegan, northern slope | 32°46'24.55" | 50°38'40.55" | 2350-2550 | 1101 | HUI |
| Esfahan, Chadegan, Kuh-e Bidak | 32°50'5.17" | 50°33'2.56" | 2200-2400 | 1357 | HUI |
| Esfahan, Zarinshahr, Hardang | 32°16'48.83" | 51°11'2.34" | 1900-2000 | 9980 | HUI |
| Shahrekord, Gardan-e Rokh | 32°20'44.58" | 51° 3'6.19" | 1250 | 4658 | HUI |
| Esfahan, 30 km Boroujen to Mobarakeh | 32° 5'2.54" | 51°26'42.31" | 2000 | 8032 | HUI |
| اEsfahan, Semirom, Hana, Cheshmeh Khuni | 31° 7'50.02" | 51°39'38.02" | 2350 | 13002 | HUI |
| Esfahan, Semirom, Kuh-e Siah | 31°25'20.48" | 51°34'35.41" | 2450 | 58237 | HUI |
| Esfahan, Shahreza, Kuh-e Dombalan | 31°45'14.24" | 51°48'10.63" |  | 17267 | HUI |
| Yasuj, 50 km east of Dehdasht, Kuh-e Neil | 30°53'56.65" | 50°50'14.33" | 2400-3200 | 46496 | HUI |
| Yasuj, between Yasuj and Dehdsht, Dilgun, Kuh-e Sarouz | 30°46'30.22" | 51° 2'18.92" | 2200-3200 | 46412 | HUI |
| Shahre Kord, between Shahre Kord and Farokh Shahr | 32°17'34.19" | 50°57'17.41" | 2100-2300 | 2775 | HUI |
| Esfahan, Semirom, Hana, Qalee Mokhtar | 31°11'52.87" | 51°47'24.30" | 2400 | 4041 | HUI |
| Esfahan, NW Esfahan, Cheshmeh Morghab | 32°51'17.12" | 50°49'3.58" | 2100 | 4175 | SFAHAN |
| Esfahan, Shahreza | 32° 2'3.13" | 51°48'10.61" | 1813 | 9922 | SFAHAN |
| Esfahan, Morghab, S Hassan Abad, Tang-e Bidekan | 32°16'22.47 | 51°24'3.04" | 1900 | 10426 | SFAHAN |
| Yasuj, Pataveh, NW Shuliz | 31 09.481 | 51 00.810 2949 | 2949 | 2681 | SFAHAN |
| Kerman, Kuh-e Hezar | 29°31'43.62" | 57°11'55.81" | 3000 | 170 | MIR |
| Kerman, Jiroft, Kuh-e Jebal Barez | 28°54'39.48" | 57°54'42.21" | 1800 | 208/1951 | MIR |
| Kerman, Joupar | 30° 0'10.54" | 57° 7'25.21" | 2350-2600 | 16188A-16183 | MIR |
| Kerman, 20 km SW Rayen, Kuh-e Hezar | 29°31'22.05" | 57°16'41.57" | 3800 | 1568 | MIR |
| Kerman, 20 km SW Rayen, Kuh-e Hezar | 29°35'21.06" | 57°20'14.19" | 2700 | 1623 | MIR |
| Kerman, Rayen, Kuh-e Hezar | 29°34'0.20" | 57°18'23.94" | 2700 | 16219 | MIR |
| Kerman, Rayen, Rayen Waterfall | 29°33'4.55" | 57°18'7.25" | 3500 | 90063 | MIR |
| Kerman, Kuh-e Lale Zar | 29°25'50.58" | 56°49'29.88" | 3800 | 76962 | MIR |
| Kerman, Kuh-e Lale Zar, southern slope | 29°28'25.43" | 56°48'36.04" | 3000 | 25199 | MIR |
| Kerman, Dalfard | 28°57'56.83" | 57°36'53.08" | 2800 | 5453 | MIR |
| Kerman, Sirch, Bololooiye | 30° 9'33.08" | 57°22'22.01" | 1600 | 398 | MIR |
| Kerman, Sarcheshmeh, close to mine | 29°57'24.98" | 55°49'34.58" | 2700 | 56424 | MIR |
| Kerman, Bam, prope Deh Bakri | 29° 2'37.87" | 57°54'5.33" | 1700-2700 | 4117 | MIR |
| Esfahan, N Semirom, Kuh-e Surmandeh | 31°31'53.69" | 51°38'50.05" | 2700-3900 | 47533 | MIR |
| Yasuj, Dena, Cheshmeh Mishi | 30°52'55.71" | 51°31'0.82" | 3200 | 2682 | Natural Resources of Kohgiluyeh and Boyer–Ahmad |
| Yasuj, Sisakht, Gardan-e Bigan | 30 53.013 | 51 31.805 | 3141 | 2679 | Natural Resources of Kohgiluyeh and Boyer–Ahmad |
| Kerman, NE Shahrbabak, Sorooshan | 30°13'36.01" | 55° 7'46.69" | 2000 | 2684 | Natural Resources of Kohgiluyeh and Boyer–Ahmad |
| Kerman, Kuhpayeh,Simak waterfall | 30°31'43.38" | 57°11'0.83" | 3200 |  | Natural Resources of Kohgiluyeh and Boyer–Ahmad |
| Yasuj, Sisakht, Gardan-e Bigan | 30°52'38.05" | 51°31'33.3" | 3220 | 7291 | Natural Resources of Kohgiluyeh and Boyer–Ahmad |
| Yasuj, Sisakht, Cheshmeh sheni | 31°13'37.54" | 51°22'24.64" | 2600 | 2048 | Natural Resources of Kohgiluyeh and Boyer–Ahmad |
| Yasuj, Shadegan village, Kuh-e khamin | 30°33'19.46" | 50°51'4.16" | 2300 | 5613 | Natural Resources of Kohgiluyeh and Boyer–Ahmad |
| Yasuj, Shadegan village, Kuh-e khamin, northern slope | 30°34'41.31" | 50°53'53.21" | 1000 | 5621 | Natural Resources of Kohgiluyeh and Boyer–Ahmad |
| Yasuj, Kuh-e Lar, southern slope | 30°34'31.61" | 50°58'12.68" | 2000 | 5640 | Natural Resources of Kohgiluyeh and Boyer–Ahmad |
| Yasuj, Kuh-e Jozar | 30°43'29.82" | 51°35'32.27" | 2300 | 5652 | Natural Resources of Kohgiluyeh and Boyer–Ahmad |
| Yasuj, between Komehr and Kakan, Maregoon village | 30°29'43.18" | 51°53'17.89" | 2200 | 74458 | Natural Resources of Kohgiluyeh and Boyer–Ahmad |
| Yasuj, Kuh-e Dena, Gardan-e Bigan | 30°52'1.88" | 51°29'40.72" | 2600 | 36-9340 | Natural Resources of Kohgiluyeh and Boyer–Ahmad |
| Yasuj, Kuh-e Dena, Gardan-e Bigan | 30°53'8.20" | 51°29'59.87" | 3500-3900 | 31238 | Natural Resources of Kohgiluyeh and Boyer–Ahmad |
| Yasuj, Sisakht, Gardan-e Bigan | 30°53'8.55" | 51°26'56.64" | 2700 | 90 | Natural Resources of Kohgiluyeh and Boyer–Ahmad |
| Yasuj, Kuh-e Dena, Gardan-e Bigan | 30°53'4.16" | 51°30'24.17" | 3300 | 12851 | Natural Resources of Kohgiluyeh and Boyer–Ahmad |
| Hormozgan, NE Bndar Abbas, Ahmadi Kousha, Bagh Chenar, | 28°12'3.44" | 56°51'23.25" | 2300 | 3183 | Animal & Natural Resources Research Center of Hormozgan |
| Hormozgan, Bndar Abbas, Kuh-e Homag Baz | 27°56'1.17" | 56°26'54.13" | 1600 | 472 | Animal & Natural Resources Research Center of Hormozgan |
| Hormozgan, Haji Abad, Kuh-e Bokoun | 28°52'40.67 | 55°43'0.87" | 3010 | 5711 | Animal & Natural Resources Research Center of Hormozgan |
| Hormozgan, Bndar Abbas, Kuh-e Homag Baz | 27°47'7.60" | 55°53'7.48" | 1700 | 507 | Animal & Natural Resources Research Center of Hormozgan |
| Khuzestan, Dehdez, Mangasht protected area | 31°43'37.75" | 50° 4'5.04" | 2500-2900 | 10635 | Natural Resources of Khuzestan |
| Khuzestan, Tight Chevilan, Kuh-e Mongar | 31° 22' 36.8" | 50° 11' 29.7" | 2700 | 8918 | Natural Resources of Khuzestan |
| Khuzestan, Kuhpayeh, Kino heighland, Kuh-e Kūh-e Leyleh | 39° 37' 29" | 49° 35' 21" | 2000 | 10678 | Natural Resources of Khuzestan |
| Khuzestan, Izeh, Kuh-e Qaroon | 31°26'16.45" | 50°17'47.08" | 1700-2600 | 2956 | Natural Resources of Khuzestan |
| Khuzestan, N Dehdez, Kuh-e Sefid | 31°52'32.36" | 50°10'49.47" | 2500 | 3051 | Natural Resources of Khuzestan |
| Khuzestan, Andika, Shimbar to Taraz road | 32°30'9.96" | 49°44'21.70" | 1900 |  | Natural Resources of Khuzestan |
| Khozestan, Andika, Bazeft valley area | 32°20'56.94" | 49°57'57.39" | 1950 |  | Natural Resources of Khuzestan |
| Khuzestan, Desfoul, After the road construction camp | 32°49'98" | 48°83'67" |  |  | Natural Resources of Khuzestan |
| Semnan, Semnan to Damghan road | 35°40'42.28" | 53°36'29.99" | 1500 | 45555 | Field |
| Semnan, 2 km north of Shahmirzad | 35°48'36.06" | 53°23'10.87" | 2100 | 82015 | Field |
| Semnan, Firuzkuh to Semnan, Gardan-e Bashm | 35°50'24.58" | 53°27'2.23" | 2600 | 40327 | Field |
| Tehran, NW Tehran, Sulqan | 35°48'19.73" | 51°15'53.37" | 1500-2000 | 57061 | Field |
| Tehran, 11km north of Abali | 35°47'27.15" | 51°57'50.88" | 2600 | 4288 | Field |
| Tehran, Damavand, Vadan | 35°36'4.47" | 52° 8'32.06" | 1800 | 54031 | Field |
| Semnan, between Mahabad and Anzeha | 35°41'13.88" | 52°40'46.61" | 2000 | 54236 | Field |
| Semnan, Firuzkuh to Damavand, Saeid Abad | 35°36'18.74" | 52°25'19.04" | 2400 | 13154 | Field |
| Semnan, Firuzkuh, Saeid Abad | 35°39'28.21" | 52°28'12.25" | 2250 | 13107 | Field |
| Semnan, Firuzkuh to Damavand, Namrood | 35°38'31.15" | 52°20'30.07" | 2200 | 22784 | Field |
| Semnan, NW Firuzkuh, Shadmahand | 35°49'30.81" | 52°32'20.83" | 2000 | 13105 | Field |
| Semnan, Damghan, Chashmeh Ali | 36°17'31.94" | 54° 3'49.06" | 1500 | 56365 | Field |
| Semnan, 71 km in Firuzkuh road, Rudehen | 35°45'8.00" | 51°53'49.40" | 220 | 7310 | Field |
| Tehran, 12km in Firuzkuh to Semnan road | 35°45'35.86" | 52°52'19.06" | 2000-2400 | 40302 | Field |
| Kerman, Kuh-e Lalezar | 29.436667 | 56.625 | 3000 |  | GBIF |
| Afghanistan, Prov. Ghorat: In jugo Kotal Tang-i Azao prope Serai Dschaura (Djaoureh) inter Qala Chahrak (Sharak) et Chisht | 34°16'0.01" | 64°25'0.00" | 2300 | 1963-0000662 | GBIF |
| Afghanistan | 34°12'0.00" | 64°17'60.00 | 2590 |  | GBIF |

**Supplementary Table1 S2.** Correlation test of climatic variables

|  | Bio 10 | Bio 12 | Bio 15 | Bio 17 | Bio 2 | Bio 3 | Bio 7 |
| --- | --- | --- | --- | --- | --- | --- | --- |
| Bio 10 | 1 |  |  |  |  |  |  |
| Bio 12 | -0.32307 | 1 |  |  |  |  |  |
| Bio 15 | 0.509647 | 0.307363 | 1 |  |  |  |  |
| Bio 17 | -0.54708 | 0.161852 | -0.42943 | 1 |  |  |  |
| Bio 2 | -0.13845 | 0.323291 | 0.453611 | -0.02555 | 1 |  |  |
| Bio 3 | 0.23624 | 0.305213 | 0.696007 | 0.081432 | 0.689658 | 1 |  |
| Bio 7 | -0.48179 | -0.01503 | -0.3698 | -0.13485 | 0.272389 | -0.4999 | 1 |
|  |  |  |  |  |  |  |  |
|  |  |  |  |  |  |  |  |

**Supplementary Table1 S3.** Percentage contribution and permutation importance of the climatic variables

| Variable | Percent contribution | Permutation importance |
| --- | --- | --- |
| Bio 12 | 64.7 | 67.2 |
| Bio 2 | 29.8 | 26.7 |
| Bio 17 | 4.6 | 3.1 |
| Bio 10 | 0.6 | 2 |
| Bio 3 | 0.3 | 1 |
| Bio 7 | 0 | 0 |
| Bio 15 | 0 | 0 |
|  | | |

**Supplementary Table1 S4.** Percentage contribution and permutation importance of the climatic variables

| Variable | Percent contribution | Permutation importance |
| --- | --- | --- |
| Bio 12 | 64.9 | 66 |
| Bio 2 | 29 | 26.9 |
| Bio 17 | 5.1 | 1.8 |
| Bio 10 | 0.6 | 3.8 |
| Bio 3 | 0.4 | 1.6 |
|  | | |


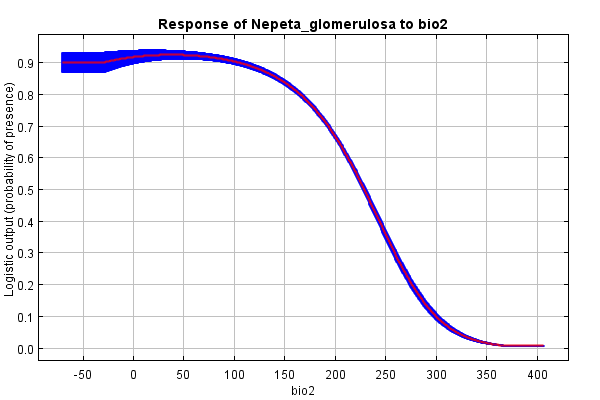

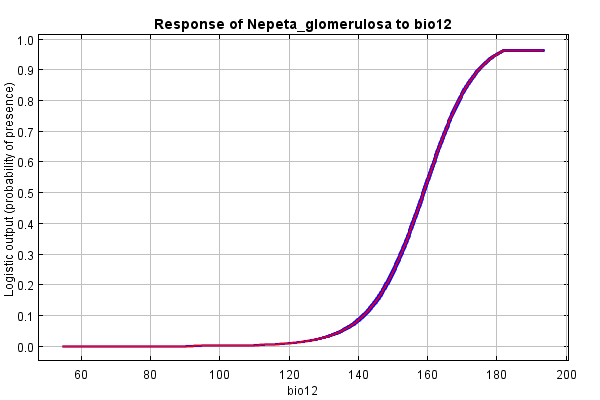


**Supplementary Fig. S1.** Response curves for the major predictors of suitability habitats of *N.glomerulosa*

z
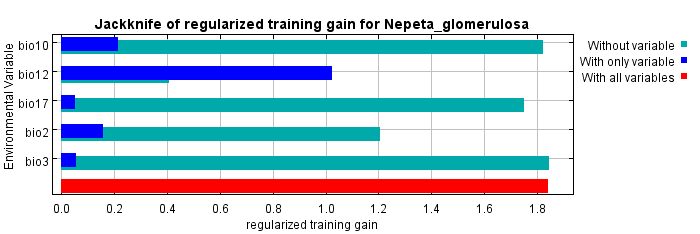


**Supplementary Fig. S2.** Results of jackknife evaluations of the relative importance of the predictor variables and their percentage contribution in *Nepeta glomerulosa* distribution.


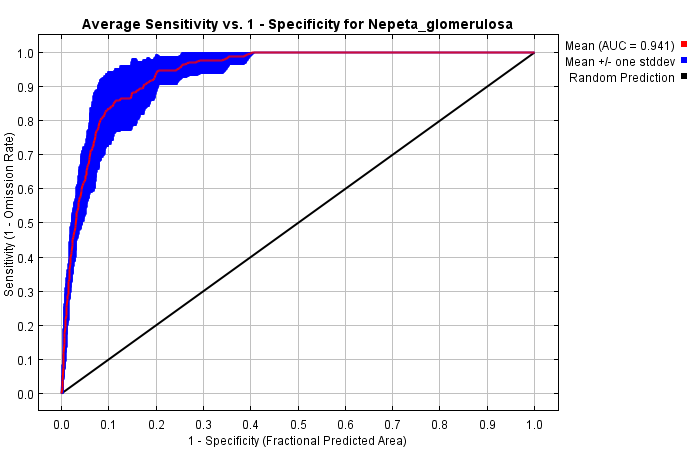


**Supplementary Fig. S3.** Results of the AUC (area under ROC) curves in developing habitat suitability model in current climate


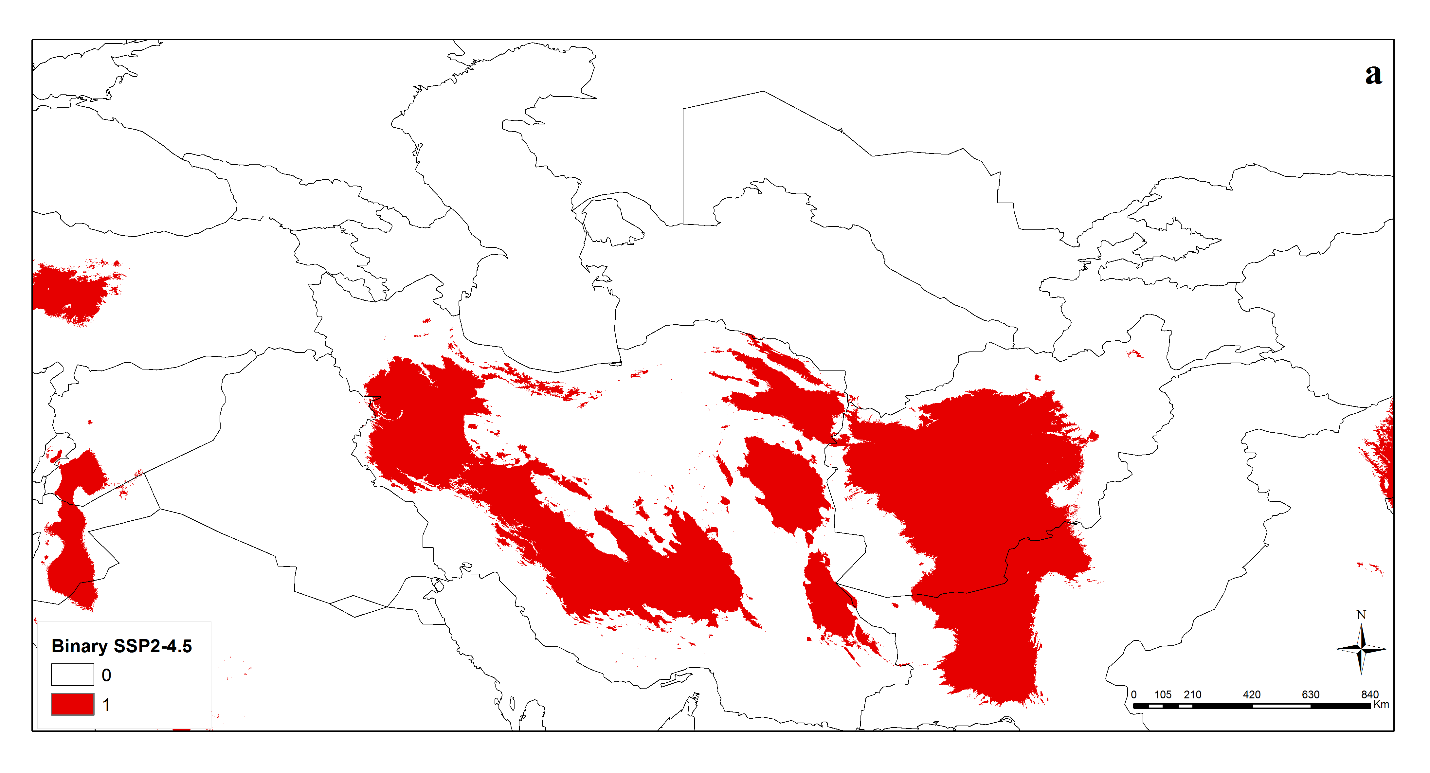

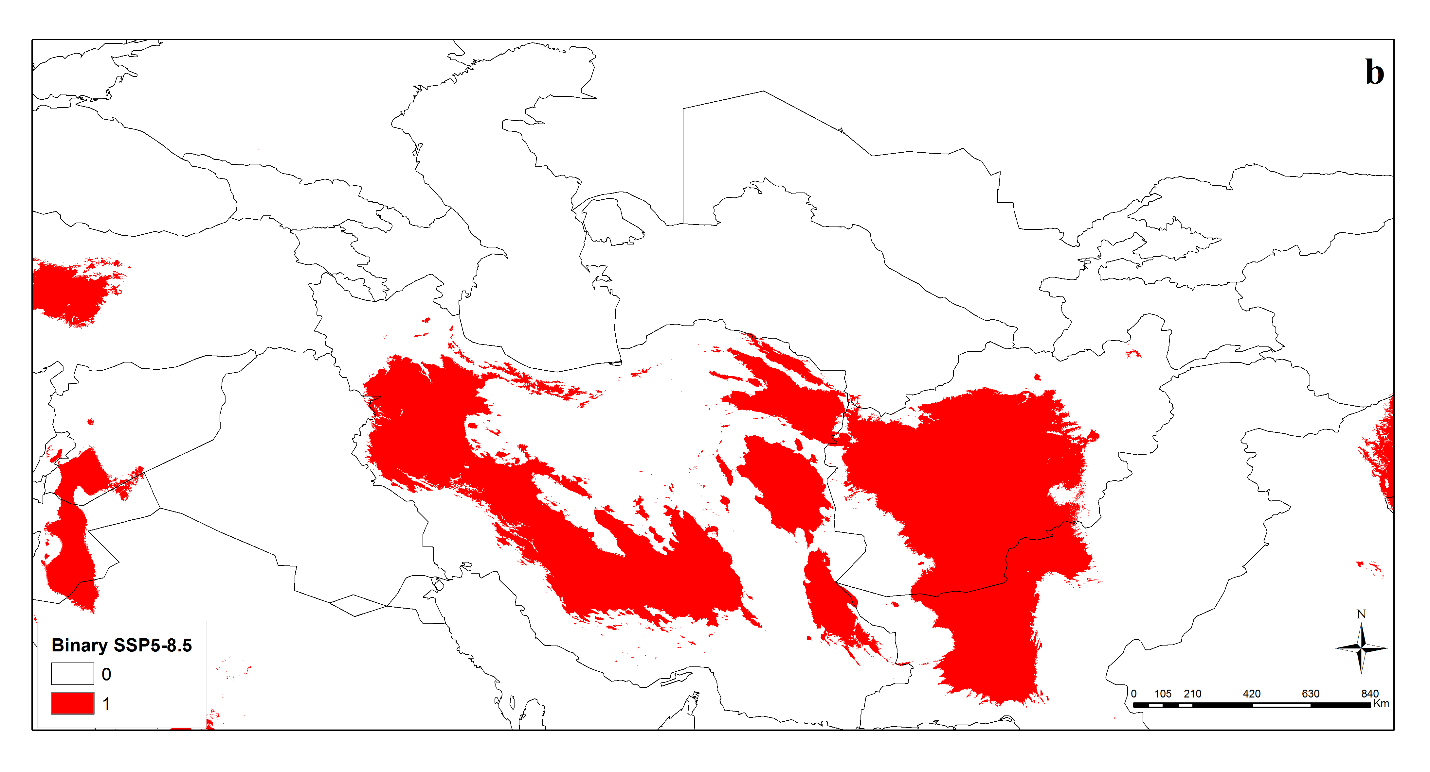


**Supplementary Fig. S4.** Potential future distribution of *Nepeta glomerulosa* under Shared Socioeconomic Pathways (SSPs) SSP2-4.5 and SSP5-8.5 across southwestern and central Asia: (a) binary map for SSP2-4.5, (b) binary map for SSP5-8.5 (from ArcMap version 10.3).
